# Microbicide Containing Ellagic Acid Can Inhibit HIV-1 Infection

**DOI:** 10.3390/molecules27227941

**Published:** 2022-11-16

**Authors:** Wipawee Nittayananta, Aornrutai Promsong, Claire Levy, Florian Hladik, Nithinart Chaitaveep, Suwipa Ungphaiboon, Supinya Tewtrakul, Surada Satthakarn

**Affiliations:** 1 Faculty of Dentistry, Thammasat University, Pathum Thani 12120, Thailand; 2 Faculty of Medicine, Princess of Naradhiwas University, Narathiwat 96000, Thailand; 3 Department of Obstetrics and Gynecology, University of Washington, Seattle, WA 98195, USA; 4 Vaccine and Infectious Disease Division, Fred Hutchinson Cancer Research Center, Seattle, WA 98195, USA; 5 Research Division, Armed Forces Research Institute of Medical Sciences, Bangkok 10400, Thailand; 6 Department of Pharmaceutical Technology, Faculty of Pharmaceutical Sciences, Prince of Songkla University, Songkhla 90110, Thailand; 7 Department of Pharmacognosy and Pharmaceutical Botany, Faculty of Pharmaceutical Sciences, Prince of Songkla University, Songkhla 90110, Thailand; 8 Department of Medical Technology, Faculty of Allied Health Sciences, Burapha University, Chonburi 20131, Thailand

**Keywords:** cytokines, chemokines, ellagic acid, HIV-1, microbicide gel

## Abstract

Objectives: Ellagic acid (EA) has a wide range of biological effects. The purpose of this study was to investigate the in vitro effects of EA on HIV-1 replication, viral enzyme activity and cytokine secretion by infected cells. Methods: The anti-HIV-1 activity of EA in solution was determined in vitro using the infection of TZM-bl cells by the nano luciferase-secreting R5-tropic JRCSF strain of HIV-1, which allows for the quantification of viral growth by measuring nano luciferase in the culture supernatants. The effect of EA on the cytokine secretion of TZM-bl cells was determined by a multiplexed bead array after 48 h of HIV-1 exposure. The antiviral effect of EA in the gel formulation (Ellagel), as would be used for vaginal application, was investigated by the inhibition of infection of UC87.CD4.CCR5 cells with R5-tropic pBaLEnv-recombinant HIV-1. Results: EA in solutions of up to 100 µM was not toxic to TZM-bl cells. EA added either 1 h before or 4 h after HIV-1 exposure suppressed the replication of R5-tropic HIV-1 in TZM-bl cells in a dose-dependent manner, with up to 69% inhibition at 50 µM. EA-containing solutions also exhibited a dose-dependent inhibitory effect on HIV-1 replication in U87 cells. When EA was formulated as a gel, Ellagel containing 25 µM and 50 µM EA inhibited HIV-1 replication in U87 cells by 56% and 84%, respectively. In assays of specific HIV-1 enzyme activity, Ellagel inhibited HIV-1 integrase but not protease. EA did not significantly modulate cytokine secretion. Conclusions: We conclude that EA either in solution or in a gel form inhibits HIV infection without adverse effects on target cells. Thus, gel containing EA can be tested as a new microbicide against HIV infection.

## 1. Introduction

As of 2021, an estimated 38.4 million people were living with human immunodeficiency virus type 1 (HIV-1), and there were an estimated 1.5 million new HIV infections globally [1]. Current treatments prevent disease progression but cannot eradicate the virus completely, and multi-drug-resistant mutants are becoming a problem [2,3,4]. Therefore, the development of novel anti-HIV therapeutics is necessary.

Medicinal plants with anti-HIV activity have been studied intensively [5,6,7,8,9]. Anti-HIV activities have been reported for xanthohumol from *Humulus lupulus* and compounds from *Rhuschinensis* [8,9], the Thai medicinal plants Hua-Khao-Yen and *Mimusopselengi* [6,7] and compounds isolated from *Boesenbergiapandurata* [5].

EA is a naturally occurring polyphenol compound found in many fruits and nuts [10]. A range of biological effects of EA have been demonstrated, including anti-allergy, anti-inflammatory, anti-bacterial [11,12] and anti-tumor properties [13,14]. EA can also modulate the expression of innate immune mediators of oral epithelial cell cultures [15]. A previous study reported that pomegranate juice containing EA can inhibit HIV entry [16], but it is unclear if EA can inhibit HIV infection in target cells post-entry. Here, we investigated the in vitro effects of EA in solution and as a gel formulation on (1) HIV enzymes in vitro, (2) HIV-1 replication and (3) cytokines produced by HIV-1-infected cells.

## 2. Results

### 2.1. EA Is Not Cytotoxic to TZM-bl Cells

**Figure 1:**
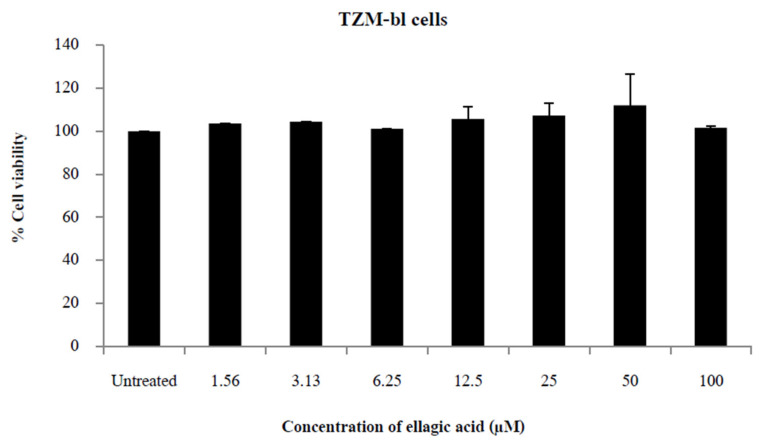
Ellagic acid cytotoxicity. Percent viability of TZM-bl cells 48 h after exposure to 1.56 to 100 μM of ellagic acid. Percent viability was measured by CellTiter-Blue assay, which used the metabolic activity of cells as a proxy for viability. Percent cell viability was calculated as: [(ODtreatedcells−ODtreatedblank)/(ODcontrolcells−ODcontrolblank)] × 100. Bars show the mean + SD of three independent experiments.

Cells were treated with various concentrations of the drug, and cytotoxicity was assessed by MTT and CellTiter-Blue assays. No cytotoxicity was observed in TZM-bl cells between 1.56 and 100 µM for 48 h (Figure 1).

### 2.2. EA in Solution Inhibits HIV-1 Replication in Target Cells

**Figure 2:**
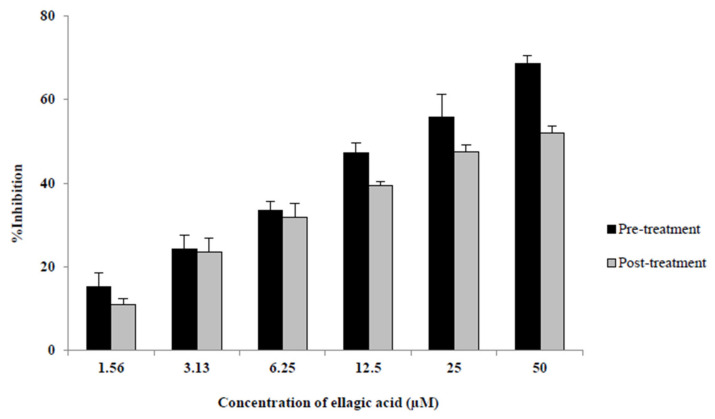
Percent inhibition of HIV-1 replication in TZM-bl cells by ellagic acid (EA). EA was added either 1 h before (“pre-treatment”) or after 4 h of exposure to HIV-1_JRCSF/NanoLuc_ and following washing off the virus (“post-treatment”). HIV replication in the cells was measured by detection of nano luciferase secreted into the cell culture supernatants using the Nano-Glo Luciferase detection assay 48 h post-challenge and was compared to HIV-1 infection without EA to calculate %Inhibition. %Inhibition = [(replication without EA−replication with EA)/replication without EA] × 100. Bars show the mean + SD of three independent experiments. All differences were significant at *p*-values of <0.05 versus the untreated control.

Cells were exposed to EA either before or after the viral challenge, and viral growth was measured by luciferase production. EA suppressed HIV replication in TZM-bl cells at 24 and 48 h post-HIV challenge when delivered either before or after HIV exposure. Compared to the untreated control, inhibition in the pre-treated cells was statistically significant and dose-dependent to a maximum of 72% at 24 h (not shown) and 69% at 48 h (Figure 2). The IC_50_ of EA pre-treatment was 11.35 µM at 24 h and 16.08 µM at 48 h. For post-treated cells, the percent inhibition of EA was also statistically significant and dose-dependent up to 53% at 24 h (IC_50_ = 29.52 µM) (not shown) and 52% at 48 h (IC_50_ = 34.11 µM) (Figure 2).

To confirm the infection, the challenged cells were assayed for β-galactosidase activity. The results were consistent with those of the Nano Luciferase assay (not shown).

**Figure 3:**
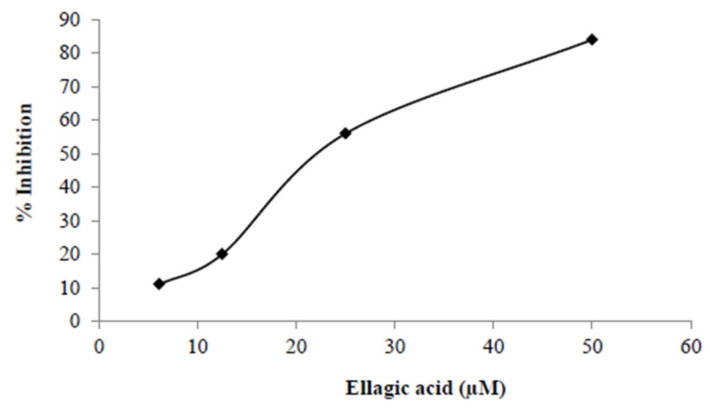
Percent inhibition of HIV-1 replication in U87CD4.CCR5 cells by ellagic acid (EA) showed a dose-dependent result. Cells were incubated for 24 h, were treated with the indicated concentrations of EA, and were incubated again for an additional hour. Then, the cells were infected with the HIV-1_JRCSF/NanoLuc_ virus and were incubated for 48 h. HIV replication in the cells was measured by detection of nano luciferase secreted into the cell culture supernatants using the Nano-Glo Luciferase detection assay 48 h post-challenge, and the treated conditions were compared to HIV-1 infection without EA to calculate %Inhibition. %Inhibition = [(replication without EA−replication with EA)/replication without EA] × 100.

Additionally, we also tested the inhibition of infection with pBaLEnv-recombinant HIV-1 in UC87.CD4CCR5 cells and found a dose-dependent inhibitory effect of EA as well (Figure 3).

### 2.3. EA Shows No Adverse Effects on Mucosal Innate Immunity

**Figure 4:**
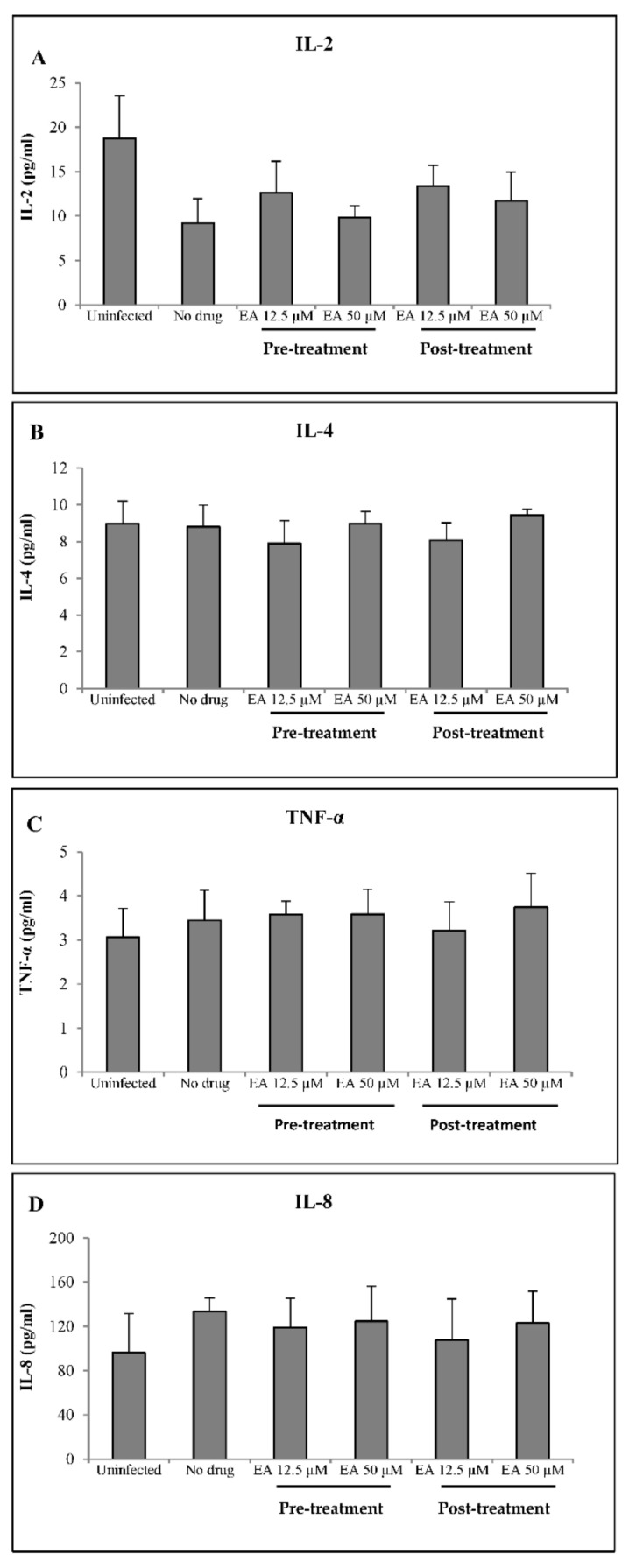

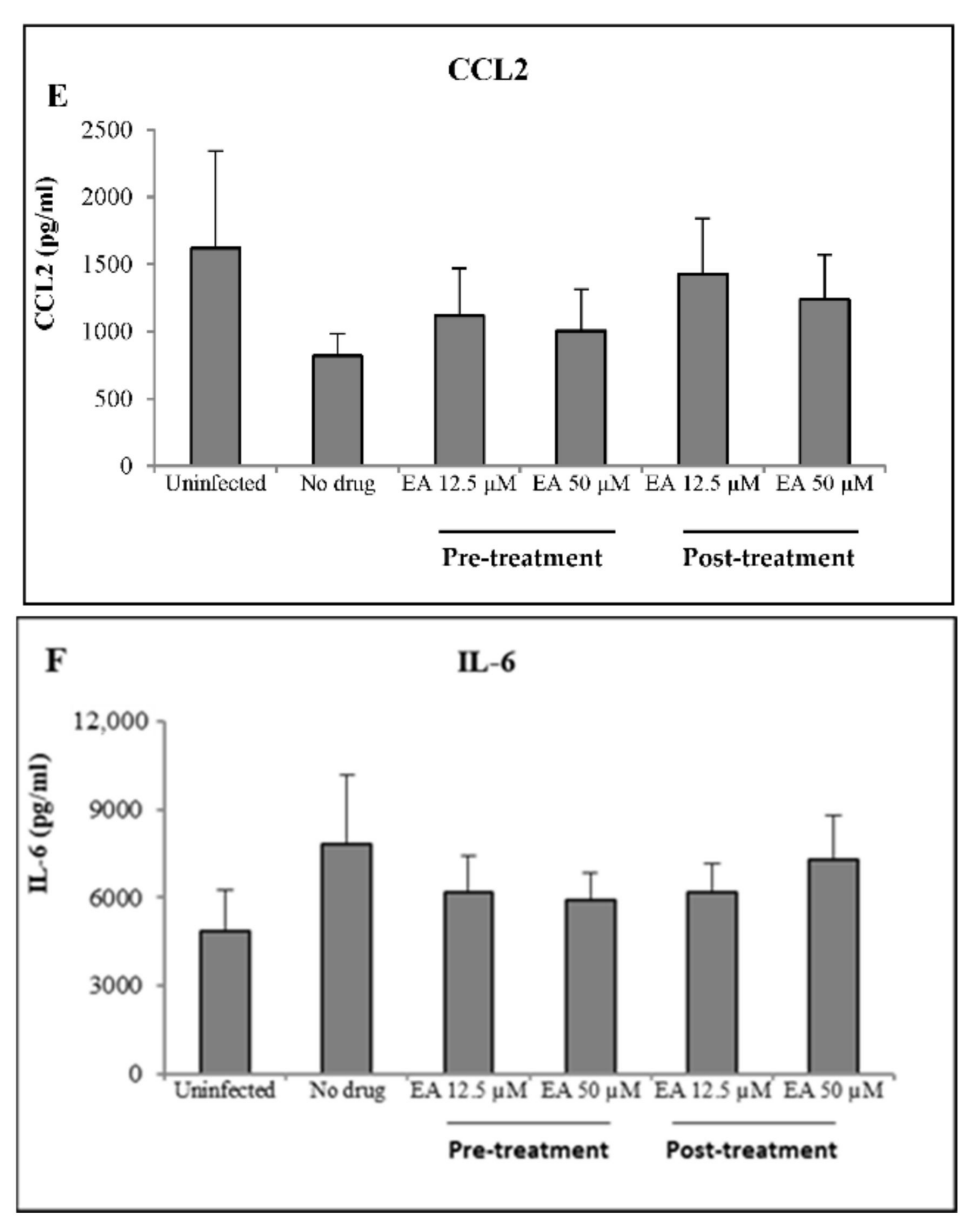
The effects of ellagic acid (EA) on levels of six immune mediators in TZM-bl cell supernatants, measured by Luminex technology at 48 h in cells treated with EA before (Pre-treatment) or after HIV-1 challenge (Post-treatment). The mediators are IL-2 (**A**), IL-4 (**B**), TNFα (**C**), IL-8 (**D**), CCL2 (**E**) and IL-6 (**F**). Bars show the mean concentration ± SD of two independent experiments. The four additional cytokines that were measured concurrently were below the level of detection and are therefore not shown.

To determine if EA affected the expression of HIV-induced innate immune mediators, supernatants were collected from TZM-bl cells exposed to HIV-1_JRCSF/NanoLuc_ in the presence or absence of EA. Six out of the ten cytokines included in the assay were detected (IL-2, IL-4, TNF-α, IL-8, CCL2 and IL-6) (Figure 4A–F). The HIV infection of TZM-bl cells corresponded with lower levels of IL-2 and CCL2 and with higher levels of IL-6 and IL-8. HIV infection did not affect IL-4 and TNF-α. The addition of EA in solution at 12.5 or 50 µM, either before or after infection, did not significantly change the levels of any detected factors.

### 2.4. Gel Containing EA Inhibits HIV-1 Replication in Target Cells

**Figure 5:**
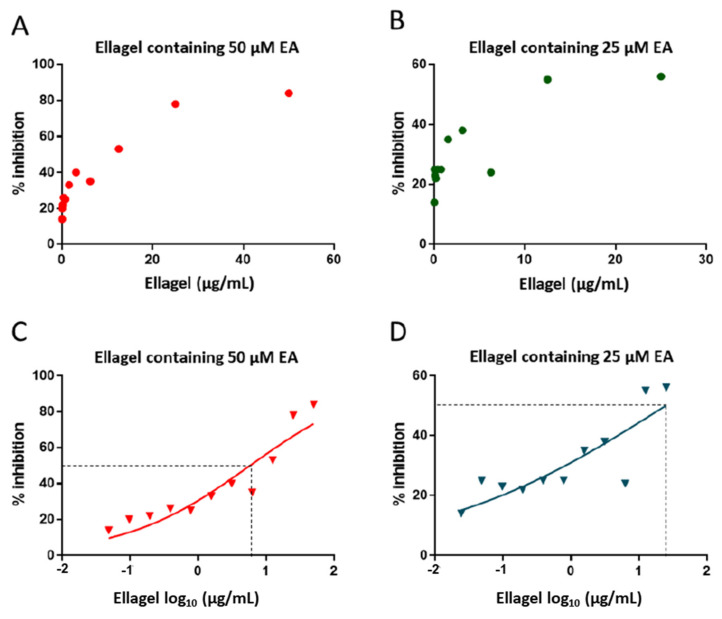
Percent inhibition of pBaLEnv-recombinant HIV-1 by Ellagel. Ellagel was formulated with either 25 μM or 50 μM of ellagic acid (EA). Then, various concentrations of Ellagel were used in the experiments, shown on the *x* axes. The replication of recombinant virus was inhibited by Ellagel 50 μM EA (red points, panels **A**,**C**) and Ellagel 25 μM EA (green points, panels **B**,**D**) in a dose-dependent manner. Panels (**C**,**D**) show the same results as (**A**,**B**) but with a log_10_ scale on the *x* axis. Dotted lines show the level of 50% inhibition of the virus (ID50). %Inhibition = [(replication without EA−replication with EA)/replication without EA] × 100.

Next, we tested the inhibitory effect of EA in the gel formulation (Ellagel). Different concentrations of Ellagel were tested for anti-HIV-1 activity. The pBaLEnv-recombinant virus was inhibited up to 84% by Ellagel containing 50 µM EA (ID_50_ = 5.79 µg/mL Ellagel) and up to 56% by Ellagel containing 25 µM (ID_50_ = 24.79 µg/mL Ellagel) (Figure 5). Low concentrations of Ellagel did not inhibit the recombinant virus (data not shown).

### 2.5. Gel Containing EA Inhibits HIV-1 Integrase but Not Protease In Vitro

**Figure 6:**
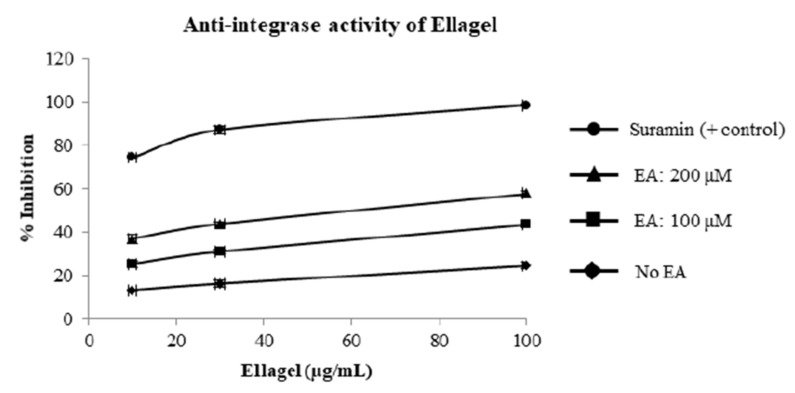
Percent inhibition of in vitro HIV integrase activity by Ellagel. Two different formulations of Ellagel, containing either 100 μM or 200 μM ellagic acid (EA), were tested. Effects of Ellagel on HIV-1 integrase activity were determined using an in vitro integration assay, in which digoxigenin-labelled target DNA was incubated with HIV-1 integrase and plate-bound HIV-long-terminal repeat (LTR) DNA in the presence or absence of Ellagel. After incubation and washing, DNA integration was detected by an alkaline phosphatase-labeled anti-digoxigenin antibody. The optical density (OD) of the AP-labeled antibody was measured at 405 nm. Anti-HIV-1 integrase activity was calculated as follows: % Anti-HIV-1 integrase activity = [(OD control − OD sample)/(OD control)] × 100. Mean % inhibition ± standard deviation of four replicates is shown in the plot.

To investigate the effect of Ellagel on HIV-1 integrase and protease, in vitro integration and proteolytic cleavage assays were performed in the presence of Ellagel. Ellagel inhibited HIV-1 integrase activity in a dose-dependent manner (Figure 6). Ellagel had no effect on HIV-1 protease activity at any of the concentrations (not shown).

## 3. Discussion

This study demonstrates that EA in solution suppressed the replication of R5-tropic HIV with a higher potency when it was added to the culture shortly before the HIV challenge compared with adding it post-challenge. EA in solution did not affect the secretion of cytokines from infected TZM-bl cells, nor was it cytotoxic. Moreover, gel formulations (Ellagel) containing between 25 and 200 µM EA effectively inhibited R5-tropic HIV-1. Together, these results suggest that EA and Ellagel possessed potent anti-HIV-1 activity without cytotoxicity and thus could serve as a novel microbicide. Anti-HIV-1 effects may occur directly via the inhibition of the integrase enzyme, as also previously reported by Promsong et al. [17], rather than indirectly via the alteration of the innate response repertoire of the infected cells.

EA may have multiple mechanisms of action against HIV infection. A previous study reported that phenolic compounds such as flavonoids inhibit HIV integrase inhibitory activity in enzyme-based assays [18], which is consistent with our findings. Flavonoids have also been reported to block the interaction between integrase and the lens epithelium-derived growth factor/p75 (LEDGF/p75), a cellular HIV-1 integration cofactor that promotes the binding of the pre-integration complex to host chromatin [18]. Promsong et al. [17] showed that EA inhibited HIV-1 integrase activity and suppressed the replication of X4-tropic HIV-1 (CRF01_AE) in C8166 cells. Another study reported that tannins prevented membrane fusion and HIV-1 entry into target cells by interfering with transmembrane glycoprotein (gp41) core formation, a critical step of viral cell fusion [19].

We also measured the effects of EA in solution on cytokines produced by HIV-infected cells. We hypothesized that EA can modulate the cellular expression of innate immune mediators in response to HIV infection, but we found no significant changes among our measured analytes. The HIV-1 infection of TZM-bl cells did have an effect; we observed decreased levels of IL-2 and CCL2 and increased levels of IL-6 and IL-8. HIV infection did not affect IL-4 and TNF-α, and the remaining four analytes were not detectable. The addition of EA, either pre-treatment or post-treatment, had a tendency toward reversing the expression of those cytokines, but this was not significant. In a separate study, we demonstrated that EA modulates innate immunity by increasing levels of RANTES and decreasing levels of IL-6 and IL-8 in oral epithelial cell culture supernatants [15]. RANTES (C_C motif ligand 5) can suppress HIV-1 infection by binding to CCR5, a host co-receptor for the R5-tropic virus [20]. The expression of IL-8 and IL-6 in HIV-1 infection is induced by HIV glycoprotein 120 (gp120), trans-activator (tat) and viral protein R (vpr) through the nuclear factor-kappa B (NF-kB) pathway [21,22,23,24]. IL-8 is a chemotactic factor for lymphocytes, andIL-6 mediates several anti-inflammatory functions [25]. A previous study demonstrated that EA down-regulates the expression of IL-6 by inhibiting NF-kB [26,27]. Although increased expression of IL-6 and IL-8 were observed in infected cells in the present study, there was no effect of EA on these cytokines.

As expected from its inhibitory effects on the HIV-1 integrase enzyme, EA inhibited HIV-1 infection in the cell lines. Consistent with these findings, other flavonoids have been reported to prevent HIV-1 infection in C8166 cells [17,18]. Thus, our results and those of other groups suggest that EA may have multiple mechanisms of action against HIV-1 infection.

A limitation of our study was the use of surrogate target cells for HIV infection rather than primary T cells or macrophages. Ellagic acid might have a weaker or stronger anti-HIV effect in these natural target cells. This limitation also applies to our assessment of the cells’ cytokine response to HIV infection and EA. Primary T cells and other leukocytes may show a different immunological response compared with TZM-bl cells. Thus, further studies are needed to fully assess the anti-HIV and immunological effects of ellagic acid. Moreover, future studies should also be performed using primary cells from the rectum, because rectal mucosa is the main route of HIV-1 infection in men who have sex with men [28]. This tissue is very transmissible, with a 10-to 2000-fold relative risk per sexual act compared to vaginal–cervical exposure [29]. Additionally, rectal mucosal tissue constitutes a key anatomical reservoir for established HIV-1 infection [30]. It is the first compartment for CD4+ T cell depletion in new infections and is rich in virus-permissive immune cells, including dendritic cells, macrophages and activated CD4+ T cells [31].

## 4. Materials and Methods

### 4.1. EA Preparation

EA was purchased as a commercial powder prepared from chestnut tree bark (Sigma, St. Louis, MO, USA). The powder was dissolved in 1 M sodium hydroxide (NaOH) to form a stock solution of 10 mM, and the final dilution was 0.03% of the initial solvent. “Ellagel”, the microbicide gel containing EA, was formulated in water with 10% polyethylene glycol 400, 3% hydroxyethyl cellulose viscosity 4000 (both Sigma-Aldrich, St. Louis, MO, USA) and various volumes of 3% ellagic acid solution, to achieve 25, 50, 100 or 200 µM of EA.

### 4.2. Cell Lines

TZM-bl is an adherent cervical epithelial cell line that expresses the HIV receptors CD4 and CCR5, as well as integrated reporter genes for firefly luciferase and β-galactosidase under the control of an HIV LTR promoter. TZM-bl cells were provided by the McElrath laboratory. TZM-bl cells were maintained in DMEM with 10% fetal bovine serum and pen/strep. U87.CD4.CCR5, a cell line that expresses CD4 and CCR5, was kindly provided by Dr. HongKui Deng and Dr. Dan R Littman and was originally obtained through the AIDS Research and Reference Reagent Program (ARRRP), Division of AIDS, NIAID, NIH. All incubations were at 37 °C with 5% CO_2_ [32,33].

### 4.3. Cytotoxicity Assay

The cytotoxicity of EA on TZM-bl cells was measured by the CellTiter-Blue assay (Promega, Madison, WI, USA) in three separate experiments. This assay measures metabolic activity as a proxy for cell viability. TZM-bl cells were grown in the presence or absence of 1.56 to 100 µM EA in triplicate and were incubated for 48 h, after which the CellTiter-blue assays were performed according to the manufacturer’s instructions, and cell viability was determined as follows:

(1) $$\%\;\mathrm{Cell}\;\mathrm{viability}=\left[\frac{\mathrm{ODtreatedcells}-\mathrm{ODtreatedblank}}{\mathrm{ODcontrolcells}-\mathrm{ODcontrolblank}}\right]\times 100$$

### 4.4. HIV-1 Constructs

The NanoLuc secreting HIV-1_JRCSF_ (HIV-1_JRCSF/NanoLuc_), a subtype B R5-tropic virus, was engineered in the McElrath Laboratory at the Fred Hutchinson Cancer Research Center. HIV-1_JRCSF/NanoLuc_ was used at 6.5 × 10^6^IFU/mL [34,35].

Non-secreted luciferase expressing pBaLEnv-recombinant virus was constructed by replacing the Env gene of pNL-Luc-EnvCT with the corresponding gene fragment of pBa-L (GenBank, Bethesda, MD, USA, accession no. AB253432), as previously described [36].

### 4.5. Exposure of TZM-bl Cells to HIV-1 Cells and EA

TZM-bl cells were exposed to HIV-1_JRCSF/NanoLuc_ at a multiplicity of infection (MOI) of 0.01 to determine if EA could inhibit HIV infection. There were four conditions:(1) pre-treatment: cells treated with EA before exposure to HIV-1; (2) post-treatment: cells treated with EA after exposure to HIV-1; (3) negative control: no EA treatment; and (4) positive control: anti-retroviral drugs (Azidothymidine at 2 µg/mL and Enfuvirtide at 10 µg/mL) added to the culture to block infection. EA was used at 1.56 to 100 µM.

For the pre-treatment conditions, cells were treated with EA for 1 h. Then, TZM-bl cells were exposed to the virus for 4 h in 1% FBS culture media containing 40 µg/mL Diethylaminoethyl-Dextran. The virus was washed off, and cells were cultured for up to 48 h to monitor infection.

For the post-treatment condition, cells were washed after 4 h of exposure to HIV-1, and the cells were cultured in the presence of EA for up to 48 h. The growth of HIV-1_JRCSF/NanoLuc_ was measured in supernatants collected at 24 h and 48 h post-HIV challenge, detecting secreted nano luciferase using the Nano-Glo Luciferase assay (Promega), according to the manufacturer’s instructions. The experiments were performed in duplicate in three separate experiments.

### 4.6. Cytokine Detection

The levels of immune mediators in supernatants from TZM-bl cells exposed to the virus in the presence or absence of EA were measured by the magnetic Human Cytokine Premixed Kit A (R&D Systems, Minneapolis, MN, USA) multiplex bead array, according to the manufacturer’s instructions. The analytes were interferon-γ (IFN-γ), interleukin-1β (IL-1β), interleukin-10 (IL-10), chemokine (C-C motif) ligand 2 (CCL2), chemokine (C-C motif) ligand 5 (CCL5) and tumor necrosis factor α (TNF-α). The plates were read on a CS 1000 Autoplex analyzer (PerkinElmer) and Luminexx MAP^TM^ Technology (Luminex Corporation, Austin, TX, USA). The experiments were performed in duplicate wells.

### 4.7. Exposure of U87.CD4.CCR5 Cells to HIV-1 and Ellagel

To investigate the effects of Ellagel on HIV-1 replication, the neutralization susceptibility of pBaLEnv-recombinant viruses to Ellagel was determined in U87.CD4.CCR5 cells [33]. A total of 5 × 10^3^U87.CD4.CCR5 cells were incubated for 24 h in 50 mL of medium. The cells were treated with 2-fold serial dilutions of Ellagel in medium and were incubated for 1 h. Then, 40 pg/mL Env-recombinant virus was added to the cultures, and the cultures were incubated for 48 h. The cells were then lysed in 100 μL of reporter lysis buffer (Passive Lysis Buffer, Promega).The supernatant from the lysate was clarified at 15,000 rpm for 5 min at 4 °C, and luciferase activity in 10 μL of clarified lysate was determined using the luciferase assay system (Promega) [37].

### 4.8. HIV-1 Enzymes Activity Assays

#### 4.8.1. HIV-1 Integrase Activity Assay

The effects of Ellagel on HIV-1 integrase activity was determined using an in vitro integration assay, in which digoxigenin-labelled target DNA was incubated with HIV-1 integrase and plate-bound HIV-long-terminal repeat (LTR) DNA, in the presence or absence of EA. After incubation and washing, DNA integration was detected by an alkaline phosphatase-labeled anti-digoxigenin antibody [7,38]. Four independent experiments were performed.

Oligonucleotides (Qiagen, Alameda, CA, USA) used for the integration assay were biotinylated LTR donor DNA (5′-biotin-ACCCTTTTAGTCAGTGTGGAAAATCTCTAGC AGT-3′/LTR-D1) and its unlabeled complement (3′-GAAAATCAGTCACACCTTTTAGAG ATCGTCA-5′/LTR-D2) and digoxigenin-labeled target substrate (TS) DNA (5′-TGACCAAGG GCTAATTCACT-digoxigenin-3′/TS-1) and its labeled complement (3′-digoxigenin-ACTGG TTCCCGATTAAGTGA-5′/TS-2). The recombinant HIV-1 integrase enzyme was grown in and purified from *Escherichia coli*.

The following buffers were prepared. The integrase buffer contained 150 mM of 3-(N-morpholino) propane sulfonic acid at a pH of 7.2, 75 mM of MnCl_2_ 2.5 mM dithiothreitol (DTT), 25% of glycerol and 500 µg/mL of bovine serum albumin. The alkaline phosphatase (AP) buffer was 100 mM of Tris-HCl (pH 9.5), 100 mM NaCl, 5 mM MgCl_2_ and 10 mM of p-nitrophenyl phosphate. Buffer-E was 20 mM 3-(N-morpholino) propane sulfonic acid, 400 mM potassium glutamate, 1 mM ethylenediaminetetraacetic acid disodium salt (EDTA-Na2), 0.1% Nonidet-P40, 20% glycerol, 1 mM DTT and 4 M urea. The integrase solution contained 1 mL of buffer-E, 1 µL of DTT and 200 µL of integrase. The washing buffer was 0.05% Tween-20 in PBS.

The 96-well plates were coated with streptavidin, followed by biotinylated-LTR DNA and 45 µL of the reaction mixture (12 µL of integrase buffer, 1 µL of digoxigenin-labelled target DNA [5 pmol/mL] and 32 µL of distilled water). Experimental wells received various concentrations of Ellagel, and only the buffer was added to negative control wells. Suramin, a polyanionic HIV-1 integrase inhibitor, was used as a positive control. Finally, 9 µL of integrase solution was added into each well, and they were incubated for 80 min to allow DNA integration.

After incubation, wells were washed four times with 300 µL PBS, and 100 µL of 500 mU/mL AP-labeled anti-digoxigenin antibody was added. The samples were incubated for 1 h, after which the wells were washed four times with washing buffer and four times with PBS.

For detection, 150 µL of AP-buffer was added to the plates, and they were incubated for 1 h. The optical density (OD) was measured at 405 nm. Anti-HIV-1 integrase activity was calculated as follows, and the results of the anti-HIV-1 integrase activity were recorded as the mean ± standard deviation of four replicates [39].

% Anti-HIV-1 integrase activity = [(OD control − OD sample)/(OD control)] × 100(2)

#### 4.8.2. HIV-1 Protease Activity Assay

The effect of Ellagel on the HIV-1 protease activity was determined with an in vitro proteolytic cleavage assay using high-performance liquid chromatography (HPLC) in three independent experiments. The reaction buffer was 50 mM sodium acetate (pH 5.0), 1 mM EDTANa2 and 2 nM 2-mercaptoethanol. The substrate peptide (Arg-Val-Nle-(pNO_2_-Phe)-Glu-Ala-Nle-NH_2_; Sigma) was diluted with 50 mM sodium acetate (pH 5.0). Recombinant HIV protease (Sigma) was diluted with the buffer and was mixed with glycerol in a 3:1 ratio. An amount of 4 µL of this HIV protease solution (0.025 mg/mL) was added to 2 µL of 5 mM sodium acetate and 2 µL of 2 mg/mL substrate peptide, yielding the reaction mixture. Reaction mixtures were prepared containing the indicated dilutions of Ellagel. The reaction mixture was incubated at 37 °C for 1 h. The reaction mixture without Ellagel was used as a negative (vehicle) control for inhibition, and acetyl pepstatin (Sigma) was used as a positive control. The reaction was stopped by heating at 90 °C for 1 min, and 20 µL of water was added to each well. Peptide cleavage was detected by HPLC using RP-18 columns (4.6 mm × 150 mm i.d., Supelco 516 C-18-DB 5 m) and acetonitrile (15–40%) and 0.2% trifluoroacetic acid in a water elution at 1.0 mL/min. The elution profile was detected at 280 nm. The substrate retention time was 11.33 min, and the p-NO_2_-Phe-bearing hydrolysate retention time was 9.47 min. The results of the anti-HIV-1 protease activity were recorded as the mean ± SD of three measurements. The HIV-1 protease inhibitory activity was calculated as follows, where A represents the relative peak area of the hydrolysate [7].

% Anti-HIV-1 protease activity = [(A control − A sample)/A control] × 100(3)

### 4.9. Statistics

The results were recorded as the mean ± SD of the duplicate or triplicate cultures of three separate experiments. The data were analyzed using a one-way ANOVA and/or the Kruskal–Wallis H test for differences between groups and were considered statistically significant at *p*-values of <0.05. The reciprocal dilution, at which viral replication was suppressed by 50% (50% inhibitory dilution, ID_50_), was calculated by the dose–response curve using a standard function of GraphPad Prism 5 software (GraphPad Software, San Diego, CA, USA).

## 5. Conclusions

In summary, this study demonstrates that ellagic acid and Ellagel can effectively inhibit HIV-1 replication, possibly through multiple molecular mechanisms, with no toxicity to the target cells.

## Data Availability

Data from this study is available upon request from the corresponding author.
